# *H*. *pylori* eradication with antibiotic treatment causes changes in glucose homeostasis related to modifications in the gut microbiota

**DOI:** 10.1371/journal.pone.0213548

**Published:** 2019-03-14

**Authors:** Gracia Mª Martín-Núñez, Isabel Cornejo-Pareja, Leticia Coin-Aragüez, Mª del Mar Roca-Rodríguez, Araceli Muñoz-Garach, Mercedes Clemente-Postigo, Fernando Cardona, Isabel Moreno-Indias, Francisco J. Tinahones

**Affiliations:** 1 Unidad de Gestión Clínica de Endocrinología y Nutrición, Instituto de Investigación Biomédica de Málaga (IBIMA), Hospital Clínico Universitario Virgen de la Victoria, Universidad de Málaga, Málaga, Spain; 2 Centro de Investigación Biomédica en Red de Fisiopatología de la Obesidad y la Nutrición (CIBEROBN), Instituto de Salud Carlos III, Madrid, Spain; 3 Departamento de Endocrinología y Nutrición, Hospital Virgen de la Victoria, Málaga, Spain; 4 UGC de Endocrinología y Nutrición, Hospital Universitario Puerta del Mar, Cádiz, Spain; Universite Paris-Sud, FRANCE

## Abstract

**Background:**

*H*. *pylori* infection and eradication cause perturbations of the gut microbiome. The gut microbiota has been identified as a potential contributor to metabolic diseases. We evaluate whether these alterations in intestinal microbiota composition produced by *H*. *pylori* infection and its posterior eradication with antibiotic treatment could be associated with glucose homeostasis in metabolically healthy subjects.

**Methods:**

Forty adult patients infected with *H*. *pylori* and 20 control subjects were recruited. The infected subjects were evaluated before and two months after eradication treatment (omeprazole, clarithromycin, amoxicillin). The microbiota composition in fecal samples was determined by 16S rRNA gene (V3-V4) sequencing using Illumina Miseq.

**Results:**

Patients (pre- and post-*H*. *pylori* eradication) showed a decreased bacterial richness and diversity with respect to controls. There was an improvement in glucose homeostasis in subjects two months after *H*. *pylori* eradication treatment. Changes in the amount of *Rikenellaceae*, *Butyricimonas*, *E*. *biforme*, *B*. *fragilis*, and *Megamonas* were inversely associated with changes in the glucose level or related parameters (Hb1ac) in *H*. *pylori* eradication subjects.

**Conclusions:**

*H*. *pylori* infection and eradication with antibiotic treatment causes alteration of the human gut microbiome. The increase in SCFA-producing bacteria and glucose-removing bacteria, specifically members of *Megamonas*, *Rikenellaceae* and *Butyricimonas*, has been related with an improvement in glucose homeostasis after *H*. *pylori* eradication with antibiotic treatment.

## Introduction

*Helicobacter pylori* is a Gram-negative bacterium that colonizes the gastric mucosa of humans and non-human primates [1]. *H*. *pylori* is typically acquired early in life and the infection often persists during patients' entire lives. The prevalence of *H*. *pylori* infection in the adult population ranges from 25–60% in Europe and up to 90% in Asia and South America, depending on geographical and infrastructural factors [2].The majority of people with *H*. *pylori* are asymptomatic and only fewer than 20% of *H*. *pylori* colonized people develop serious diseases (e.g. multifocal atrophic gastritis, gastric adenocarcinoma, mucosa-associated-lymph-tissue [MALT] lymphoma) [3–4].

*H*. *pylori* infection is associated with modifications in the gastric microenvironment and in the composition of the indigenous gastric microbiota [5], but might also trigger large intestinal microbiota changes leading to a new physiological gastrointestinal balance [6]. While there are no studies in humans, some animal studies have reported changes in the gut microbiota after *H*. *pylori* infection [7–9].Proton pump inhibitor-based therapy with two antibiotics is the treatment of choice for *H*. *pylori* eradication, which causes perturbation of the gut microbiome in humans [1, 10–11].Some studies have confirmed the induction of long-term disturbances in the intestinal microbiota from the eradication therapy [10–11].In contrast, changes in the microbiota during *H*. *pylori* eradication reverted to normal soon after treatment was completed [11].Alterations to the microbiome caused by infection, diet, antibiotics and/or lifestyle can disturb this symbiotic relationship and promote diseases including type 2 diabetes and obesity, among others [8, 12]. Previous studies have associated *H*. *pylori* infection and eradication with lipid and glucose metabolism [13–14]. In this context, changes in the intestinal microbiota induced by *H*. *pylori* infection and antibiotic eradication treatment could be a significant contributor to the development of metabolic disorders. While several animal studies have associated alterations of the gut microbiota by *H*. *pylori* infection with glucose homeostasis [8–9], to the best of our knowledge, there are no studies in humans that relate changes in the gut microbiota profile of patients with *H*. *pylori* infection and after the eradication treatment to glucose metabolism. Thus, we hypothesize that both infection and the eradication treatment of *H*. *pylori* may cause perturbations in the gut microbiome, which can indirectly affect carbohydrate homeostasis.

## Materials and methods

### Study population and design

Forty consecutive adults infected by *H*. *pylori*, were screened and recruited from the Microbiology Department through positive *H*. *pylori* stool antigen immunochromatography assay. Sample size was assessed considering a reduction in richness of 16% because of the antibiotic therapy based on previous microbiota studies [15–17] and a pilot study (non-published). Sample size resulted in 35 subjects for the intervention study. Thus, 40 consecutive patients were selected who met the following inclusion criteria:1) age range 18–65 years, and 2) with their first *H*. *pylori* infection. Moreover, a control group of healthy participants (20 participants) matched by age, gender and dyspeptic symptoms, but negative for *H*. *pylori* stool antigen was also studied. Exclusion criteria were established for 1) diagnosis of type 1 or type 2 diabetes; 2) prior documented treatment of *H*. *pylori*; 3) antibiotic use within the three months previous to enrollment; 4) informed consent could not be obtained. Diabetic subjects were excluded from the study, because both diabetes and its treatment have been associated with specific changes in gut microbiota [18–19], which could negatively interfere with the objectives of the present study.

The study included two visits, one prior to and one two months after antibiotic eradication treatment (omeprazole 20mg, clarithromycin 500mg, amoxicillin 1000mg twice daily for 10 days), for patients and only one visit for the control group. Patients with negative *H*. *pylori* stool antigen immunochromatography assay two months after the antibiotic treatment were selected for this study. All visits included a physical examination, a dietary survey, a fasting blood sample, and a 75g oral glucose tolerance test (OGTT) at 30, 60, and 120 minutes. Also, fecal samples were collected during each visit and stored at -80°C until DNA extraction.

The study protocol was approved by the Medical Ethics Committee at Virgen de la Victoria University Hospital and conducted in accordance with the Declaration of Helsinki. Written informed consent was provided by all participants, who were also verbally informed of the characteristics of the study.

### Anthropometric, biochemical and dietetics measurements

Body weight, height, and waist circumferences were measured according to standardized procedures [20]. Serum glucose after fasting and OGTT at time points 30, 60 and 120 minutes after 75 g of glucose, total cholesterol, high-density lipoprotein (HDL)cholesterol, triglycerides (Randox Laboratories Ltd) and C-reactive protein (Dimension autoanalyzer; Dade Behring Inc.) were measured using a standard enzymatic method. Low-density lipoprotein (LDL) cholesterol was calculated using the Friedewald formula. Insulin was analyzed by immunoradiometric assay (BioSource International). Glycosylated hemoglobin (HbA_1c_) (%) was measured using a high performance liquid chromatography method in a Variant Turbo autoanalyzer (Bio-Rad).

The variable area under the glucose curve (AUC) was calculated from serum glucose concentrations at different time points obtained in the oral glucose tolerance test by the trapezoidal rule and presented as total AUCs. The insulin resistance index was calculated according to the homeostasis model assessment (HOMA-IR) [21] and pancreatic beta-cell function was estimated by the HOMA (HOMA-B) using the following equation: [fasting plasma insulin (microunits per milliliter) X 20] / FBG (millimolars) - 3.5.

Both total energy (kcal / day) and macronutrients (proteins, fats, total carbohydrates, dietary fiber and sugars (g / day)) and micronutrients (total polyphenols (mg / day)) for each participant were obtained from 24-hour dietary recalls for 7 days, using DIAL nutrition program and the professional Diet Balancer software (Cardinal Health Systems Inc.).

### Microbial diversity analysis

#### DNA extraction

Fresh fecal samples were immediately frozen at −80°C after collection and kept until use. Stool DNA was extracted from stool samples using the QIAamp DNA Stool Mini Kit, according to the manufacturer's protocols (Qiagen, Germany). Stool DNA concentrations were measured using a Qubit Fluorometric (Thermo Fisher Scientific).

#### 16S rRNA gene amplification by PCR

The fecal bacterial microbiota composition was determined using tag-encoded16S rRNA gene Miseq-based (Illumina, CA, USA) high-throughput sequencing. The 16S rRNA V3-V4 amplicon (amplicon size ~460bp) was amplified by polymerase chain reaction (PCR) (95°C for 3 min, followed by 25 cycles at 95°C for 30 s, 55°C for 30 s, and 72°C for 30 s and a final extension at 72°C for 5 min) using the universal primers reported by Klindworth et al. [22] fused with Illumina adapter overhang nucleotide sequences. Primer sequences were 5'TCGTCGGCAGCGTCAGATGTGTATAAGAGACAGC[CTACGGGNGGCWGCAG] -3’ and 5'GTCTCGTGGGCTCGGAGATGTGTATAAGAGACAG-[GACTACHVGGGTATCTAATCC]-3’. Each 25 μL of polymerase chain reaction (PCR) reaction holds 12.5ng of fecal genomic DNA as template, 12.5 μL of Master Mix (2x KAPA HiFiHotStartReady Mix) and 5 μL of 1μM of each primer.The PCR products were checked using electrophoresis in 2% (w/v) agarose gels. A bioanalyzer (Agilent 2100, USA) was used to verify the size of the PCR product.

#### 16S gene library construction, quantification, and sequencing

AMPure XP beads (Beckman Coulter Genomic, CA, USA) were used to purify the free primers and primer dimer species in the amplicon product. Dual indices and Illumina sequencing adapters were attached to sequence the amplicons, using the Nextera XT Index Kit (Illumina, CA, USA) and purified the amplicon again using AMPure XP beads (Beckman Coulter Genomic, CA, USA). Before sequencing, DNA concentration of each PCR product was determined using a Qubit Fluorometric double-stranded DNA assay (Thermo Fisher Scientific) and Bioanalyzer DNA 1000 chip to verify the size (Agilent 2100, USA). The amplicons from each reaction mixture were pooled in equimolar ratios based on their concentration. The sample pool (4nM) was denatured and diluted following Illumina guidelines. Paired-end sequencing of amplicons was conducted on the Illumina MiSeq platform using the v3 kit generating 2 × 301 nucleotide reads (Illumina, San Diego, USA).

#### Bioinformatic analysis

The merged paired-end reads were analyzed using the Quantitative Insights into Microbial Ecology (QIIME) tool (version 1.9.1; open source software) [23]. Operational taxonomic units (OTUs) were picked by the conservative script pick_closed_reference_otus.py against the Greengenes 16S rRNA gene database (gg13_8) at a similarity of 97% by submitting each cluster to UCLUST in order to obtain the taxonomy assignment and the relative abundance of each OTU. Alpha diversity (microbial diversity within samples) and beta diversity (community diversity between samples) analyses were performed using QIIME. Alpha diversity analyses were computed for rarefied OTU tables (set to 85% of the sequence number within the most indigent sample, corresponding to 34,385 sequences) using the alpha rarefaction workflow. The alpha diversity was estimated using Chao1 and Shannon indexes. Beta diversity was calculated through beta_diversity_through_plots.py on even subsampled OTU table, with the default beta diversity metrics of weighted and unweighted UniFrac distance matrices [24] which (were used to perform Principal Coordinate Analysis (PCoA) to determine the similarity between groups of samples.

### Statistical analysis

The statistical analysis was performed with SPSS 22.0 (SPSS Inc., Chicago, IL, USA) and QIIME (version 1.9.1; open source software).The data were expressed as mean ± standard deviation. In order to check changes in the relative abundance (%) of operational taxonomic units (OTUs) and in clinical, biochemical and anthropometric variables between groups, the Wilcoxon’s signed-rank test (for paired samples) and the Mann-Whitney U test (for independent samples) were used, whereas to check qualitative changes in OTUs (presence/absence) between groups, the G-test of independence (g_test) was used. Alpha diversity between different groups of the samples was assessed by non-parametric two-sample t-test (compare_alpha_diversity.py). The analysis of similarity (ANOSIM) statistical test was performed via QIIME (compare_categories.py—method anosim) to test the statistical significance between groups. Spearman’s correlation coefficient was also used to examine the relationship between the OTUs and biochemical variables. A multivariate regression analysis was performed to identify individual changes in OTUs as independent predictors for changes in the AUC and HbA_1c_ levels. For regression and correlation analysis, the variables AUC and HbA_1c_ were expressed as % of change, as well as the OTU differential. Statistical significance was set at *P*<0.05, reported by the conservative false discovery rate (FDR)-corrected p-value for multiple comparisons or p-value, as appropriate.

## Results

### Anthropometric and biochemical characteristics

The anthropometric and biochemical variables of the patients before and after *H*. *pylori* eradication treatment, as well as those of the control subjects, are depicted in Table 1.

**Table 1:** Anthropometric and biochemical variables.

Variables	Pre-*H*. *pylori* eradication (n = 40) (1)	Post-*H*.*pylori* eradication (n = 40) (2)	Controls (n = 20) (3)	p value (1–3)*	p value (1–2)*	p value (3–2)*
Age(years)	46.95±12.78	46.95±12.78	43.86±12.63	NS	NS	NS
Men /women (n)	16/24	16/24	9/13	**—**	**—**	**—**
BMI (kg/m^2^)	26.92**±**4.30	26.91**±**4.40	25.89**±**4.54	NS	NS	NS
Waist (cm)	92.10**±**12.06	91.27**±**11.73	89.8**±**13.23	NS	NS	NS
Fasting plasma glucose (mg/dL)	93.72**±**7.56	93.47**±**7.60	90.60**±**11.07	NS	NS	NS
Fasting plasma insulin (μUI/ml)	8.28 **±** 6.11	8.63**±**6.11	8.08**±**4.97	NS	NS	NS
HOMA-IR	1.96±1.6	2.03±1.5	1.89±1.3	NS	NS	NS
HOMA-β	95.38±59.2	100±56.87	102±47.08	NS	NS	NS
HbA_1c_ (%)	5.44**±**0.50	5.28**±**0.36	5.29**±**0.30	NS	0.005	NS
HDL cholesterol (mg/dL)	52.97**±**12.9	55.36**±**16.36	57**±**15.8	NS	0.044	NS
LDL cholesterol (mg/dL)	121.45**±**35.8	117.96**±**33.4	102.05**±**34	0.036	NS	0.07
Triglycerides (mg/dL)	97.2**±**39.6	93.5**±**36.4	89.70**±**41.78	NS	NS	NS
Cholesterol (mg/dL)	194.22**±**40.84	191.34**±**37.15	177.05**±**39.5	NS	NS	0.08
DBP (mmHg)	77.75±9.58	80.50±11.37	75.95±10	NS	0.08	NS
SBP (mmHg)	123.84±16.62	125.42±21.36	120.3±13.35	NS	NS	NS
CRP (mg/L)	4.07±2.44	3.56±2.11	4.14±2.92	NS	NS	NS

All values are means **±**standard deviations. Wilcoxon’s signed-rank test was used in comparing before and after *H*. *pylori* eradication. The Mann-Whitney U test was used to compare the unpaired-samples.

*P-value for the comparison of the variables between different groups (1, 2, 3).

NS: p>0.05. BMI: Body mass index, HbA_1c_: Glycosylated Hemoglobin, LDL: Low-density lipoprotein, HDL: High-density lipoprotein, DBP: Diastolic blood pressure, SBP: Systolic blood pressure, CRP: C-reactive protein.

*H*. *pylori* patients and control subjects were balanced according to age and sex. No differences were found in anthropometric parameters such as BMI and waist circumference or biochemical parameters such as glucose, insulin, HOMA.IR, HOMA-β, triglycerides, and cholesterol levels. However, the HDL-cholesterol level significantly increased after *H*. *pylori* eradication therapy, while the LDL-cholesterol level was lower in controls than in patients before *H*. *pylori* eradication treatment.

Regarding the dietary assessment, no statistically significant differences were observed in the comparisons of micronutrients and macronutrients between patients and controls (p> 0.05) (data not shown).

### Patients after *H*. *pylori* eradication treatment improves glucose homeostasis

**Fig 1:**
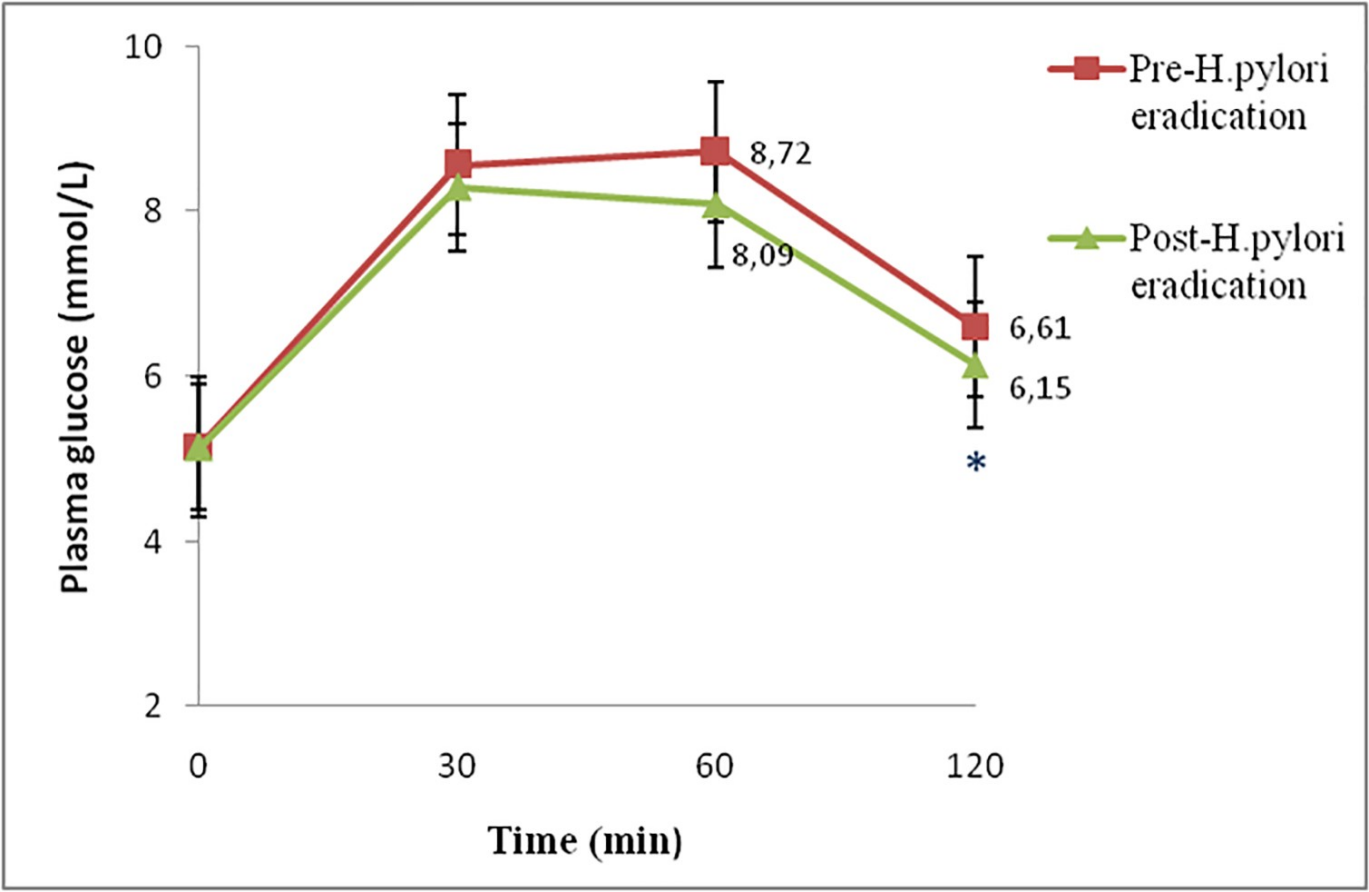
Glucose tolerance curve in patients before and after *H*. *pylori* eradication. The asterisks indicate p< 0.05. Glucose values (mmol/L) are shown for p <0.05.

Fasting plasma glucose concentrations and HbA_1c_ are shown in Table 1, while the postprandial plasma glucose profiles for pre and post *H*.*pylori* eradication patients are depicted in Fig 1.Whereas plasma glucose concentrations at baseline and 30 minutes post-ingestion of a glucose bolus were similar from 60 minutes an improvement in glucose metabolism was observed, meaning a decrease in glucose levels, in post-*H*. *pylori* eradication patients (p = 0.01) (Fig 1). There were no significant differences between patients and controls. Glucose levels at 120 minutes differed significantly from baseline for patients and controls (p≤0.01). Thus, patients after *H*. *pylori* eradication treatment showed a decrease in the AUC and in HbA_1_ levels with respect to patients before *H*. *pylori* eradication treatment (874.15**±**249.06 vs. 917.61**±**249.6; p = 0.006; and 5.28±0.36 vs. 5.44±0.50, p = 0.005, respectively).There were no statistically significant differences in AUC and HbA_1c_ between patients with infection and controls (917.61±249.6 vs. 899.79±190.8 and 5.44±0.50 vs. 5.29±0.30, respectively).

### *H*. *pylori* eradication treatment affects gut microbiota diversity

A total of 13,747,554 high-quality sequences and 59,614 OTUs were obtained from the total samples, although samples were rarefied to 34,208 sequences per sample, which corresponded to 85% of the lowest number of quality reads obtained from any individual sample in the dataset. Moreover, in order to increase the statistical power, OTUs that were not found in at least five different samples, were excluded from the analysis. These reads/OTUs were assigned to 12 phyla, 49 families, 75 genera and 42 different species.

**Fig 2:**
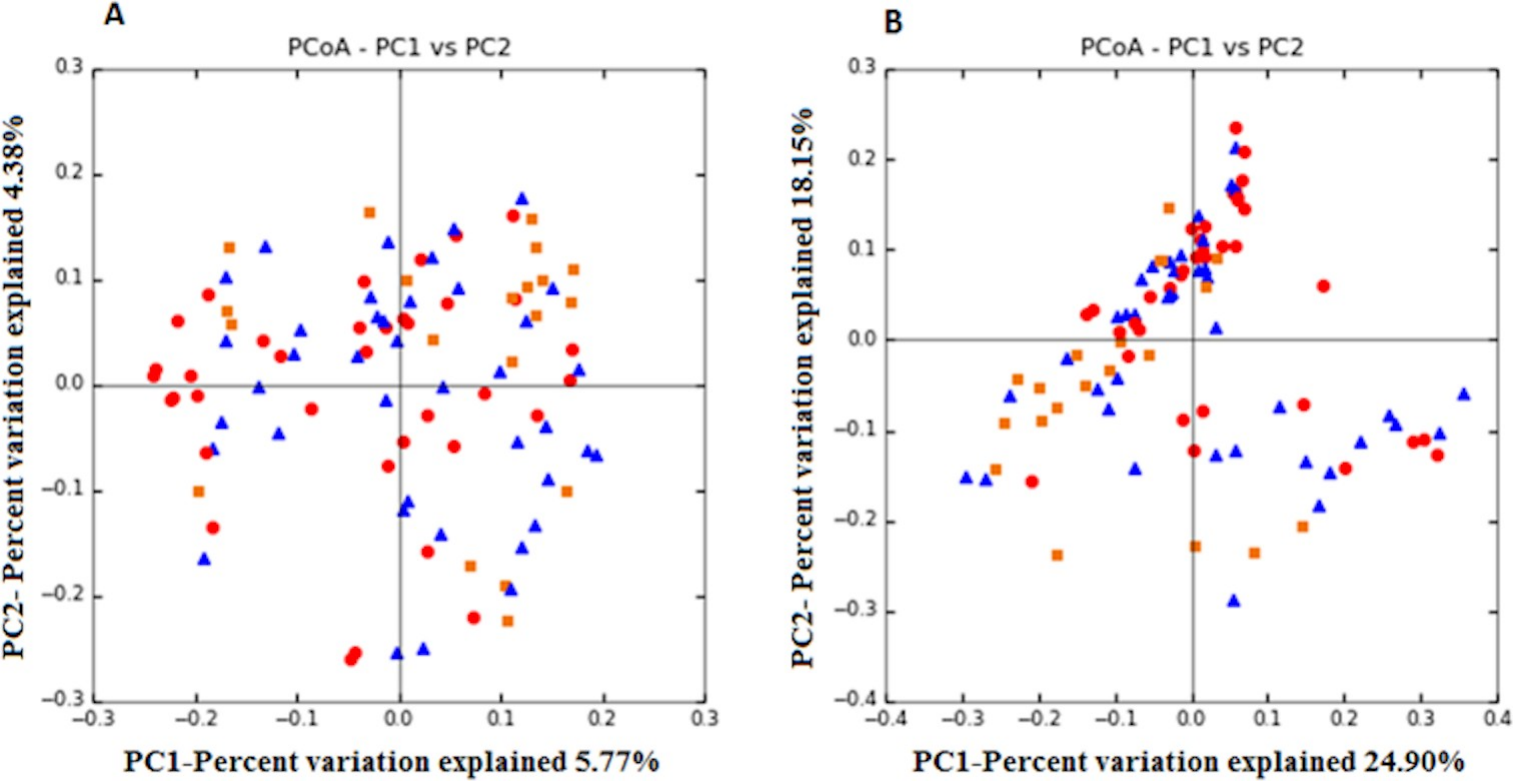
**Clustering of fecal bacterial communities according to the different study groups by principal coordinate analysis (PCoA) using unweighted (A) and weighted (B) UniFrac distances.** Each point corresponds to a community coded according to the patient group and control group. The percentage of variation explained by the plotted principal coordinates is indicated on the axes. Orange square: control group; blue triangle: pre-eradication group; red dot: post-eradication group.

In order to visualize complex relationships, Dimensional Principal Coordinates Analysis plots of unweighted and weighted Unifrac distances were used to assess the similarity of microbial communities between the studied groups (Fig 2).The ANOSIM statistical test confirmed that fecal communities from control subjects and pre- and post-treatment *H*. *pylori* patients differed significantly (unweighted Unifrac, ANOSIM test, p = 0.01). Taking into account the abundance of the bacteria, a better explanation was observed as the percentage of variance explained was higher (weighted Unifrac,ANOSIM test, p = 0.01), indicating a clear effect of this factor on the ecological diversity of the groups. Delving further into the results, no differences were observed between the control and pre-treatment groups and pre- and post-treatment patients (weighted ANOSIM, ANOSIM test, p>0.05), while a significant difference was found between the control and post-treatment fecal communities (weighted Unifrac, ANOSIM test, p = 0.01), indicating a clear influence of the antibiotic treatment for *H*. *pylori* on the fecal ecology of the patients (S1 Fig).

Alpha diversity assessment using rarefaction curves revealed clear differences among the studied groups, estimated by the indexes of Chao1 (Richness) and Shannon (Diversity).As expected, control subjects showed the greatest diversity and richness, showing statistical differences with respect to the *H*. *pylori* patients (pre- and post-eradication treatment). Within the *H*. *pylori* patients, the eradication treatment affected richness (p = 0.041), indicating a decrease in the number of registered OTUs, and evenness was almost statistically significant (p = 0.051) (Table 2).

**Table 2:** Estimates of alpha diversity in control subjects and patients before and two months after *H*. *pylori* eradication.

	Pre-*H*. *pylori* eradication (n = 40) (1)	Post-*H*. *pylori* eradication (n = 40) (2)	Controls (n = 20) (3)	*p-value (3–1)	*p-value (1–2)	*p-value (3–2)
Chao1	3280.47**±**707.04	2941.22**±**710.71	3979.51**±**808.089	0.002	0.041	0.001
Shannon	6.11**±**0.58	5.83**±**0.66	6.49**±**0.52	0.017	0.051	0.001

All values are means **±** standard deviations.

* P-value obtained for comparison of the richness and diversity index between different groups (1, 2, 3).

### Gut microbiota profile is clearly different after *H*. *pylori* eradication treatment

**Fig 3:**
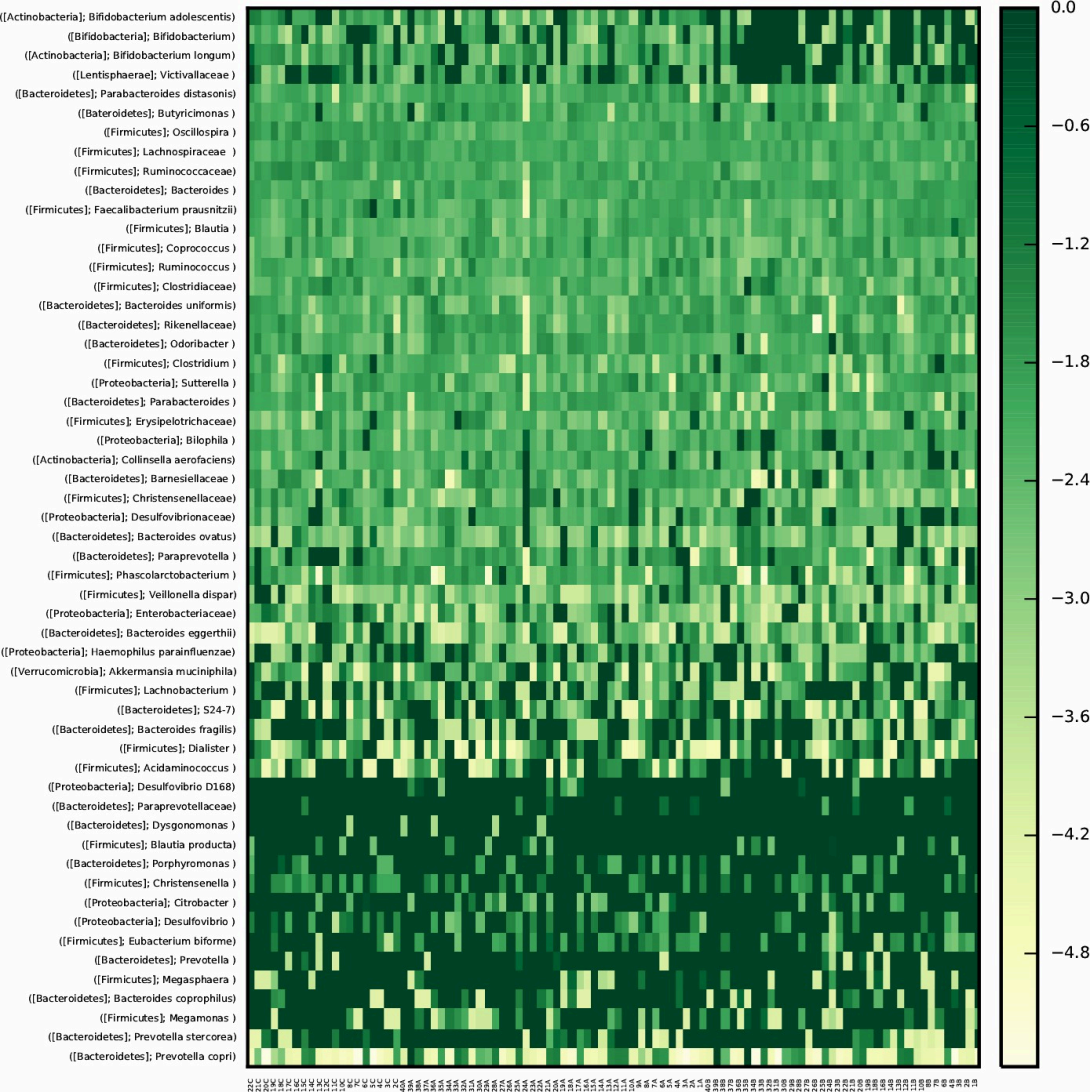
Presence/Absence microbiota heatmap of the study groups. Significant bacterial taxa among groups according to the likelihood-ratio test (G-test) are depicted (P<0.05, raw and -FDR-corrected).

According to the qualitative assessment of the OTU discovered, a different occurrence (presence/absence) is depicted (Fig 3). Due to the high number of changes found between the groups, only those OTUs found to be statistically different (p<0.05) between patients before and after *H*. *pylori* eradication treatment and associated significantly with the variables of our study (AUC and HbA_1c_) are highlighted. In this manner, we observed a lower presence of the *Rikenellaceae* family, the *Butyricimonas* genus and *E*. *biforme* and a greater presence of *B*. *Fragilis* and *Megamonas* genus in patients after *H*. *pylori* eradication treatment.

**Fig 4:**
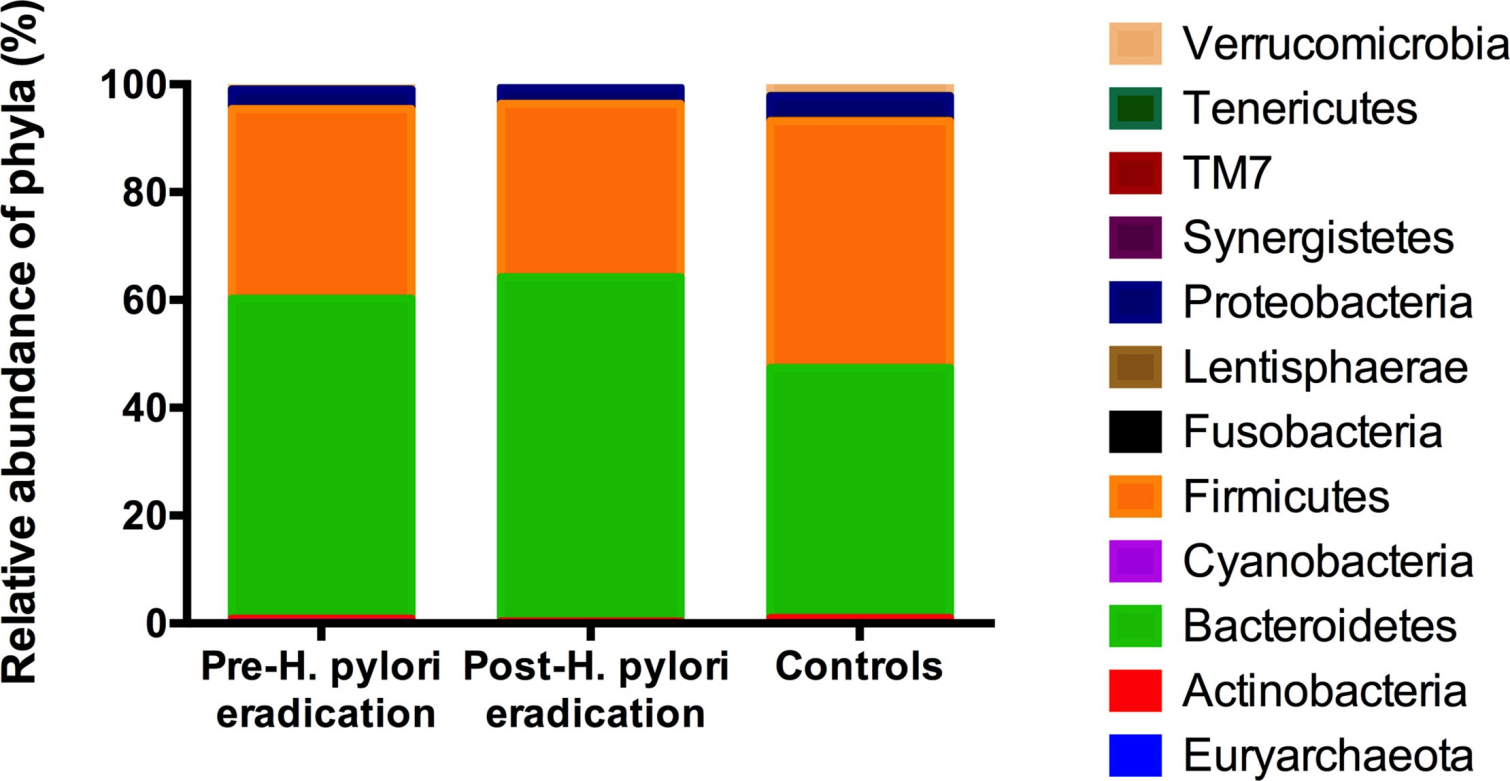
Mean relative abundance of bacterial phyla (%) in the controls and patients before and two months after *H*. *pylori* eradication.

Regarding the relative abundance of each OTU in the fecal samples collected, the dominant bacterial phyla were, as expected, *Firmicutes* and *Bacteroidetes*. *Actinobacteria*, *Proteobacteria* and *Verrucomicrobia* contributed smaller proportions, between 1–5% (Fig 4). *Bacteroidaceae* was the predominant family followed by *Ruminococcaceae*, *Lachnospiraceae*, *Prevotellaceae* and *Veillonellaceae* (>5%) (S2 Fig), while the dominant genera were *Bacteroides*, *Prevotella* and *Parabacteroides* (>5%). *Paraprevotella*, *Lachospira*, *Oscillospira*, *Dialister*, *Phascolartobacterium*, *Ruminonococcus*, *Sutterella*, and *Akkermansia* contributed lower proportions, between 1–5% (S3 Fig).With respect to the species level, *F*.*prausnitzii*, *P*.*copri*, *P*.*distasonis*, and *B*.*uniformis* were the most abundant (>1%).

Significant changes in relative abundance comparing controls and patients before and after *H*. *pylori* eradication treatment were found in our study (S4 Fig). *Bacteroidetes* was the most representative phylum among the *H*. *pylori* patients at pre- and post-eradication time points (58.72±13.62% and 63.50±10.30%, respectively), while the *Bacteroidetes* and *Firmicutes* phyla remained at similar levels in the control subjects (45.89±13.57% vs. 45.68±15.61%, p = 0.82) (Fig 4). In this manner, the *Bacteroidetes*/*Firmicutes* ratio did not significantly differ between patients before and after *H*. *pylori* eradication treatment (p<0.05), with greater values than control subjects in both cases (2.03±1.28 vs. 1.26±0.91 and 2.29±1.16 vs. 1.26±0.91, respectively, p≤0.005). Moreover, we found a decrease in the relative abundance of *Actinobacteria* post-*H*. *pylori* eradication compared with pre-*H*. *pylori* eradication (0.27±0.41% vs. 0.77±0.96%, p = 0.001) and controls (0.27±0.41% vs. 0.97±1.2%, p = 0.004).

On the other hand, within the *Actinobacteria* phylum, we found that the *H*. *pylori* eradication treatment led to a significant decrease in the relative abundance of the family *Bifidobacteriaceae*, and especially in the genus *Bifidobacterium* and *B*. *longum* and *B*. *adolescentis* species. *Firmicutes* and *Proteobacteria* phyla also experimented a decrease after the *H*. *pylori* eradication treatment, specifically a decrease in the relative abundance of the family *Streptococcaceae* and the genus *Streptococcus*. Moreover, after the eradication treatment a decrease in the abundance of *Turicibacteraceae* and the genera *Turicibacter*, *Ruminococcaceae* and Oscillospira, as well as the family *Oxalobacteriaceae* and the genus *Oxalobacter* and *O*. *Formigenes* species, and the family *Enterobacteriaceae* were reported with respect to the control group (Table 3).

**Table 3:** Comparison of relative abundance of families, genera and species between controls and patients before and two months after *H*. *pylori* eradication treatment within the phyla Actinobacteria, Firmicutes, Proteobacteria.

Phyla	Families/Genera/ Species	Pre-*H*. *pylori* eradication (n = 40) (1)	Post-*H*. *pylori* eradication (n = 40) (2)	Controls (n = 20) (3)	p* (1–3)	p* (1–2)	p* 3–2)
Actinobacteria	*Bifidobacteriaceae*	0.44±0.63	0.12±0.38	0.72±1.2	NS	0.0003	0.0006
	*Bifidobacterium*	0.45±0.63	0.12±0.38	0.72±1.12	NS	0.0005	0.001
	*B*. *Longum*	0.096±0.19	0.022±0.04	0.17±0.31	NS	0.014	0.0017
	*B*. *Adolescentis*	0.21±0.13	0.19±0.58	0.25±0.30	NS	0.0002	0.0009
Firmicutes	*Streptococcaceae*	0.52**±**0.78	0.11**±**0.18	0.16**±**0.20	NS	0.017	NS
	*Streptococcus*	0.52±0.78	0.11±0.18	0.16±0.19	NS	0.03	NS
	*Turicibacteraceae*	0.01±0.02	0.008±0.02	0.02±0.07	NS	NS	0.022
	*Turicibacter*	0.01±0.02	0.008±0.02	0.03±0.07	NS	NS	0.04
	*Ruminococcaceae*	11.66**±**5.26	11.04**±**6.51	18.90**±**8.1	0.055	NS	0.020
	*Oscillospira*	1.02±0.66	1.28±0.78	1.98±1.12	0.02	NS	NS
Proteobacteria	*Oxalobacteriaceae*	0.03**±**0.04	0.02**±**0.06	0.05**±**0.08	NS	NS	0.028
	*Oxalobacter*	0.03±0.04	0.02±0.06	0.06±0.08	NS	NS	0.04
	*O*. *Formigenes*	0.03±0.03	0.02±0.05	0.06±0.07	NS	NS	0.053
	*Enterobacteriaceae*	0.53**±**1.13	0.22**±**0.47	1,40**±**1,84	NS	NS	0.028

Values are means **±** standard deviations; Wilcoxon’s signed-rank test was used in comparing pre- and post-*H*. *pylori* eradication. The Mann-Whitney U test was used to compare unpaired-samples.

*P value obtained for comparison of relative abundance (%) between the different groups (1, 2, 3).NS: p>0.05 (false discovery rate post hoc test).

### Modifications in glucose metabolism are associated to bacterial changes

In order to establish a possible relationship between the glucose metabolism status of the studied groups and their gut microbiota profiles, correlation studies were performed. Significant univariate correlations were found between changes in the amount of specific bacteria and the proportion of changes in the glucose AUC (*Rikenellaceae*: r = -0.45, p = 0.005; *Butyricimonas*: r = -0.39, p = 0.017 and *E*.*biforme*: r = -0.33, p = 0.044), as well as with the proportion of HbA_1c_ changes (*B*. *Fragilis*: r = -0.36, p = 0.03 and *Megamonas*: r = -0.38, p = 0.02) in patients after *H*. *pylori* eradication treatment.

Multivariate regression analyses, for all the bacterial groups analyzed, were assessed. Only the changes in *Rikenellaceae* (R^2^ = 0.086, β = -0.33, p = 0.04 and R^2^ = 0.130, β = -0.33, p = 0.04) and *Butyricimonas* (R^2^ = 0.133, β = -0.397, p = 0.016 and R^2^ = 0.273, β = -0.510, p = 0.002) predicted the proportion of changes in the glucose AUC in patients after the eradication treatment. These results remained statistically significant even after correcting for age, sex and BMI.

## Discussion

In this study, we have shown that *H*. *pylori* eradication with antibiotic treatment produces specific bacterial changes associated with an improvement in glucose homeostasis and HbA_1c_ levels in patients with normal blood glucose concentrations. HbA_1c_ is an index of long-term glycemic control and a risk predictor used in the monitoring of diabetes. However, HbA_1c_ levels are acquiring a big relevance also in apparently healthy subjects [25] because of its successful standardization among subjects. Our study has shown moderate changes in HbA1c between patients before and after the H. pylori eradication. However, these variations in HbA_1c_ are statistically significant and could be clinically relevant; by analogy with other studies, these small changes are similar to those observed after life-style modifications, for example, after dietary interventions and physical exercise [26–27]. The favorable effect of *H*. *pylori* eradication on glucose homeostasis have been reported in previous studies [28–29]. However, the mechanisms underlying the association between *H*. *pylori* eradication and glucose homeostasis are unclear. We propose that gut microbiota mediated, at least partially, this improvement in the glucose homeostasis.

*H*. *pylori* has been reported to interact with gastric microbiota [5], whereas there is scarce literature regarding its association with gut microbiota [7–9]. Our data have shown, for the first time, changes in the gut microbial profile associated with *H*. *pylori* infection in humans, while several studies have confirmed that the antibiotic treatment used in *H*. *pylori* eradication affects the gut microbiota [1, 10–11]. In this line, we have shown that the common 10-day antimicrobial treatment with clarithromycin, amoxicillin and omeprazole decreases the diversity and richness of gut microbiota, and that these effects are persistent even two months after *H*. *pylori* eradication treatment. This indicates that antibiotic-induced microbiota alterations can remain after long periods of time [11, 30], without the total recovery of the initial state [30].

In our study, these changes in the intestinal microbiota after antibiotic treatment were the result of the significant increases in *Bacteroidetes* and decrease in *Firmicutes*, accompanied by important decreases within other phyla, such as *Actinobacteria* and *Proteobacteria*. These data indicate that the broad-spectrum antibiotics used in *H*. *pylori* eradication treatment are capable of inhibiting a huge range of bacteria [1, 10, 31]. However, some bacteria benefitted from this change in the intestinal ecosystem. Indeed, *Megamonas* showed superior levels, even than the control subjects, two months after antibiotic administration. Exposure of the colonic ecosystem to antimicrobial agents disturbs the initial ecological niche facilitating the colonization of specific members in the gut microbiota [32].

Alterations in gut microbiota profile have been related to deterioration in metabolic health [12, 33]. Low bacterial richness has been characterized by a more marked overall adiposity, insulin resistance and dyslipidemia and a more pronounced inflammatory phenotype [34], as well as alterations in the bile acid metabolism [35]. However, according to other studies [36–38], we have observed an amelioration of glycemia after antibiotic treatment in spite of the reduction in gut microbiota diversity. Indeed, we have been the first ones relating specific changes in gut bacteria with a metabolic amelioration after *H*. *pylori* eradication treatment. Particularly, we have found an inverse association between *Rikenellaceae*, *Butyricimonas*, *E*. *biforme*, *B*. *fragilis*, *Megamonas* and glucose levels (AUC) or related parameters (HbA_1c_) after treatment.

Several studies have shown that these bacteria, with a special mention to *Megamonas Rikenellaceae* and *Butyricimonas*, are involved in the fermentation of non-digestible carbohydrates and generation of short-chain fatty acids (SCFAs) such as acetate, propionate and butyrate [39–42]. Previous studies have linked these SCFAs with the host metabolism, and especially with glucose metabolism. In fact, butyrate-producing bacteria have been related to an improvement of the glucose tolerance in association with decreased endotoxemia [43], as well as with an amelioration in insulin sensitivity [44–45]; propionate induces intestinal gluconeogenesis, through the gut–brain neural circuit, improving peripheral glucose production and insulin sensitivity [46]; and acetate could also act on the parasympathetic activity increasing food intake and promoting glucose-stimulated insulin secretion [47].Other studies have suggested that the binding of SCFAs to GPR43 and GPR41 increases the plasma levels of glucagon-like peptide-1 (GLP-1) and peptide YY (PYY), leading to an improved glucose homeostasis and reduced appetite [48]. However, few studies have associated these bacteria with glucose homeostasis [49]. But, more interestingly, *Rikenellaceae* and *Butyricimonas* members are also able to use the environmental glucose for this SCFAs production, helping to regulate glucose levels [41–42]. In this regard, our data have related, for the first time, changes *Rikenellaceae* and *Butyricimonas* with the prediction of glucose proportions. Moreover, Rikenellaceae, Butyricimonas explained 8% and 13%, respectively, of the changes observed in AUC. These results could indicate that the loss of diversity and richness produced by the antibiotic therapy is not as important as the loss or gain of the function that these organisms may play.

In the present study, there are several limitations that must be taken into consideration. The 16S ribosomal RNA gene sequencing used has limitations in identifying genetically specific species and strains as well as little information on bacterial genes and their functions. On the other hand, sample size could be augmented, although previous sample size calculations were done ensuring a realistic approach. Another limitation of the study was, the lack of group of subjects without an H. pylori infection exposed to the eradication treatment due to ethical reasons. These data could have provided more detailed information on the role of antibiotic treatment in the association found. Moreover, microbial metabolites data could help to support our results, which will have taken into account for future experiments.

Importantly, these findings suggest that compositional changes in the gut microbiota produced by *H*. *pylori* eradication with antibiotic treatment could be related, with the glucose homeostasis of the host. The involvement of glucose-removing bacteria such as *Rikenellaceae* and *Butyricimonas*, as well as the increase SCFA-producing bacteria as *Megamonas*, could play a role in this association. These findings may be useful for developing strategies for the improvement of glucose homeostasis in subjects with a glucose imbalance by modulation of the abundance of specific taxa, such as those discovered in this study: mainly *Megamonas*, *Rilenellaceae* and *Butyricimonas*. Next steps could be to validate these associations in independent cohorts and to prove a possible causal axis between these bacteria and glucose homeostasis in functional studies.

## Supporting information

S1 Fig **Clustering of fecal bacterial communities according to the different study groups by principal coordinate analysis (PCoA) using unweighted (A) and weighted (B) UniFracdistances.** Each point corresponds to a community coded according to the patients and controls group: **1)** Pre- (blue dot) vs. Post-*H*. *pylori* eradication (red squared),**2)** Controls(redsquared) vs. Post-*H*. *pylori* eradication (red dot). **3)** (bluesquared) vs. Pre-*H*. *pylori* eradication (red dot).The percentage of variation explained by the plotted principal coordinates is indicated on the axes.
(TIF)

S2 Fig Mean relative abundances (%) of bacterial families in the controls and patients before and 2 months after *H*. *pylori* eradication. Other: sequences unassigned to OTU
(TIF)

S3 Fig Mean relative abundances of bacterial (%) genera in the controls and patients before and 2 months after H. pylori eradication. Other: sequences unassigned to OTU.
(TIF)

S4 Fig Abundance microbiota heatmap of the study groups. **Significant bacterial taxa among groups are depicted.** Wilcoxon’s signed-rank test was used in comparing pre and post-*H*. *pylori* eradication. U de Mann-Whitney was used to compare the unpaired-samples. Moreover, significant taxa after a multiple FRD correction (P<0.05) are indicated as: [a]: Pre- vs. Post-*H*. *pylori* eradication; [b]: control vs. Pre-H. Pylori eradication; [c]: Control vs. Post-*H*. *pylori* eradication.
(TIFF)

## Abbreviations

AUC: Area Under the Glucose Curve
Hba1c: Glycosylated Hemoglobin
HDL: High Density Lipoprotein
LDL: Low density lipoprotein
OTUs: Operational Taxonomic Units
OGTT: Oral Glucose Tolerance Test
SCFA: Short Chain Fatty Acid

## References

[1] Yap TW, Gan HM, Lee YP, Leow AH, Azmi AN, Francois F, et al Helicobacter pylori Eradication Causes Perturbation of the Human Gut Microbiome in Young Adults. PLoS One. 2016;11(3):e0151893 Epub 2016/03/19. 10.1371/journal.pone.0151893 PONE-D-15-55436 [pii]. 26991500 PMC4798770

[2] Goh KL, Chan WK, Shiota S, Yamaoka Y. Epidemiology of Helicobacter pylori infection and public health implications. Helicobacter. 2011;16 Suppl 1:1–9. Epub 2011/09/16. 10.1111/j.1523-5378.2011.00874.x 21896079 PMC3719046

[3] Logan RP. Helicobacter pylori and gastric cancer. Lancet. 1994;344(8929):1078–9. Epub 1994/10/15. . 7934456 10.1016/s0140-6736(94)91729-9

[4] Malfertheiner P, Chan FK, McColl KE. Peptic ulcer disease. Lancet. 2009;374(9699):1449–61. Epub 2009/08/18. S0140-6736(09)60938-7 [pii] 10.1016/S0140-6736(09)60938-7 . 19683340

[5] Maldonado-Contreras A, Goldfarb KC, Godoy-Vitorino F, Karaoz U, Contreras M, Blaser MJ, et al Structure of the human gastric bacterial community in relation to Helicobacter pylori status. ISME J. 2011;5(4):574–9. Epub 2010/10/12. ismej2010149 [pii] 10.1038/ismej.2010.149 20927139 PMC3105737

[6] Lopetuso LR, Scaldaferri F, Franceschi F, Gasbarrini A. The gastrointestinal microbiome—functional interference between stomach and intestine. Best Pract Res Clin Gastroenterol. 2014;28(6):995–1002. Epub 2014/12/03. S1521-6918(14)00155-3 [pii] 10.1016/j.bpg.2014.10.004 . 25439066

[7] Heimesaat MM, Fischer A, Plickert R, Wiedemann T, Loddenkemper C, Gobel UB, et al Helicobacter pylori induced gastric immunopathology is associated with distinct microbiota changes in the large intestines of long-term infected Mongolian gerbils. PLoS One. 2014;9(6):e100362 Epub 2014/06/19. 10.1371/journal.pone.0100362 PONE-D-13-05285 [pii]. 24941045 PMC4062524

[8] He C, Yang Z, Cheng D, Xie C, Zhu Y, Ge Z, et al Helicobacter pylori Infection Aggravates Diet-induced Insulin Resistance in Association With Gut Microbiota of Mice. EBioMedicine. 2016;12:247–54. Epub 2016/10/26. S2352-3964(16)30417-0 [pii] 10.1016/j.ebiom.2016.09.010 27743904 PMC5078605

[9] Gao XX, Ge HM, Zheng WF, Tan RX. NMR-based metabonomics for detection of Helicobacter pylori infection in gerbils: which is more descriptive. Helicobacter. 2008;13(2):103–11. Epub 2008/03/07. HEL590 [pii] 10.1111/j.1523-5378.2008.00590.x . 18321300

[10] Jakobsson HE, Jernberg C, Andersson AF, Sjolund-Karlsson M, Jansson JK, Engstrand L. Short-term antibiotic treatment has differing long-term impacts on the human throat and gut microbiome. PLoS One. 2010;5(3):e9836 Epub 2010/03/31. 10.1371/journal.pone.0009836 20352091 PMC2844414

[11] Buhling A, Radun D, Muller WA, Malfertheiner P. Influence of anti-Helicobacter triple-therapy with metronidazole, omeprazole and clarithromycin on intestinal microflora. Aliment Pharmacol Ther. 2001;15(9):1445–52. Epub 2001/09/13. apt1033 [pii]. . 11552917 10.1046/j.1365-2036.2001.01033.x

[12] Moreno-Indias I, Cardona F, Tinahones FJ, Queipo-Ortuno MI. Impact of the gut microbiota on the development of obesity and type 2 diabetes mellitus. Frontiers in microbiology. 2014;5:190 Epub 2014/05/09. 10.3389/fmicb.2014.00190 24808896 PMC4010744

[13] Buzas GM. Metabolic consequences of Helicobacter pylori infection and eradication. World J Gastroenterol. 2014;20(18):5226–34. Epub 2014/05/17. 10.3748/wjg.v20.i18.5226 24833852 PMC4017037

[14] Roca-Rodriguez MM, Coin-Araguez L, Cornejo-Pareja I, Alcaide J, Clu-Fernandez C, Munoz-Garach A, et al Carbohydrate metabolism improvement after Helicobacter pylori eradication. Diabetes Metab. 2016;42(2):130–4. Epub 2015/12/30. S1262-3636(15)00160-3 [pii] 10.1016/j.diabet.2015.11.003 . 26712113

[15] Moreno-Indias I, Sanchez-Alcoholado L, Garcia-Fuentes E, Cardona F, Queipo-Ortuno MI, Tinahones FJ. Insulin resistance is associated with specific gut microbiota in appendix samples from morbidly obese patients. Am J Transl Res. 2016;8(12):5672–84. Epub 2017/01/13. 28078038 PMC5209518

[16] Moreno-Indias I, Sanchez-Alcoholado L, Perez-Martinez P, Andres-Lacueva C, Cardona F, Tinahones F, et al Red wine polyphenols modulate fecal microbiota and reduce markers of the metabolic syndrome in obese patients. Food & function. 2016;7(4):1775–87. Epub 2015/11/26. 10.1039/c5fo00886g . 26599039

[17] Murri M, Leiva I, Gomez-Zumaquero JM, Tinahones FJ, Cardona F, Soriguer F, et al Gut microbiota in children with type 1 diabetes differs from that in healthy children: a case-control study. BMC Med. 2013;11:46 Epub 2013/02/26. 1741-7015-11-46 [pii] 10.1186/1741-7015-11-46 23433344 PMC3621820

[18] Leiva-Gea I, Sanchez-Alcoholado L, Martin-Tejedor B, Castellano-Castillo D, Moreno-Indias I, Urda-Cardona A, et al Gut Microbiota Differs in Composition and Functionality Between Children With Type 1 Diabetes and MODY2 and Healthy Control Subjects: A Case-Control Study. Diabetes Care. 2018;41(11):2385–95. Epub 2018/09/19. dc18-0253 [pii] 10.2337/dc18-0253 . 30224347

[19] Wu H, Esteve E, Tremaroli V, Khan MT, Caesar R, Manneras-Holm L, et al Metformin alters the gut microbiome of individuals with treatment-naive type 2 diabetes, contributing to the therapeutic effects of the drug. Nat Med. 2017;23(7):850–8. Epub 2017/05/23. nm.4345 [pii] 10.1038/nm.4345 . 28530702

[20] Loham T RA, Martorel R,. Standardization of anthropometric measurements. Human Kinetics. 1988.

[21] International Expert Committee report on the role of the A1C assay in the diagnosis of diabetes. Diabetes Care. 2009;32(7):1327–34. Epub 2009/06/09. dc09-9033 [pii] 10.2337/dc09-9033 19502545 PMC2699715

[22] Klindworth A, Pruesse E, Schweer T, Peplies J, Quast C, Horn M, et al Evaluation of general 16S ribosomal RNA gene PCR primers for classical and next-generation sequencing-based diversity studies. Nucleic Acids Res. 2013;41(1):e1 Epub 2012/08/31. gks808 [pii] 10.1093/nar/gks808 22933715 PMC3592464

[23] Caporaso JG, Kuczynski J, Stombaugh J, Bittinger K, Bushman FD, Costello EK, et al QIIME allows analysis of high-throughput community sequencing data. Nat Methods. 2010;7(5):335–6. Epub 2010/04/13. nmeth.f.303 [pii] 10.1038/nmeth.f.303 ; PubMed Central PMCID: PMC3156573. 20383131 PMC3156573

[24] Lozupone C, Knight R. UniFrac: a new phylogenetic method for comparing microbial communities. Appl Environ Microbiol. 2005;71(12):8228–35. Epub 2005/12/08. 71/12/8228 [pii] 10.1128/AEM.71.12.8228-8235.2005 16332807 PMC1317376

[25] Saha S, Schwarz PEH. Impact of glycated hemoglobin (HbA1c) on identifying insulin resistance among apparently healthy individuals. J Public Health. 2017;25:505–12. 10.1007/s10389-017-0805-4

[26] Church TS, Blair SN, Cocreham S, Johannsen N, Johnson W, Kramer K, et al Effects of aerobic and resistance training on hemoglobin A1c levels in patients with type 2 diabetes: a randomized controlled trial. JAMA. 2010;304(20):2253–62. Epub 2010/11/26. 304/20/2253 [pii] 10.1001/jama.2010.1710 21098771 PMC3174102

[27] Barnard ND, Cohen J, Jenkins DJ, Turner-McGrievy G, Gloede L, Green A, et al A low-fat vegan diet and a conventional diabetes diet in the treatment of type 2 diabetes: a randomized, controlled, 74-wk clinical trial. Am J Clin Nutr. 2009;89(5):1588S–96S. Epub 2009/04/03. ajcn.2009.26736H [pii] 10.3945/ajcn.2009.26736H PubMed Central PMCID: PMC2677007. 19339401 PMC2677007

[28] Dogan Z, Sarikaya M, Ergul B, Filik L. The effect of Helicobacter pylori eradication on insulin resistance and HbA1c level in people with normal glucose levels: a prospective study. Biomed Pap Med Fac Univ Palacky Olomouc Czech Repub. 2015;159(2):242–5. Epub 2014/07/06. 10.5507/bp.2014.036 . 24993741

[29] Gen R, Demir M, Ataseven H. Effect of Helicobacter pylori eradication on insulin resistance, serum lipids and low-grade inflammation. South Med J. 2010;103(3):190–6. Epub 2010/02/06. 10.1097/SMJ.0b013e3181cf373f . 20134372

[30] Jonsson M, Qvarnstrom Y, Engstrand L, Swedberg G. Clarithromycin treatment selects for persistent macrolide-resistant bacteria in throat commensal flora. Int J Antimicrob Agents. 2005;25(1):68–74. Epub 2004/12/29. S0924-8579(04)00347-4 [pii] 10.1016/j.ijantimicag.2004.08.011 . 15620829

[31] Peters DH, Clissold SP. Clarithromycin. A review of its antimicrobial activity, pharmacokinetic properties and therapeutic potential. Drugs. 1992;44(1):117–64. Epub 1992/07/01. 10.2165/00003495-199244010-00009 . 1379907

[32] Nord CE. The effect of antimicrobial agents on the ecology of the human intestinal microflora. Vet Microbiol. 1993;35(3–4):193–7. Epub 1993/06/01. . 8212506 10.1016/0378-1135(93)90144-v

[33] Karlsson FH, Tremaroli V, Nookaew I, Bergstrom G, Behre CJ, Fagerberg B, et al Gut metagenome in European women with normal, impaired and diabetic glucose control. Nature. 2013;498(7452):99–103. Epub 2013/05/31. 10.1038/nature12198 . 23719380

[34] Le Chatelier E, Nielsen T, Qin J, Prifti E, Hildebrand F, Falony G, et al Richness of human gut microbiome correlates with metabolic markers. Nature. 2013;500(7464):541–6. Epub 2013/08/30. nature12506 [pii] 10.1038/nature12506 . 23985870

[35] Vrieze A, Out C, Fuentes S, Jonker L, Reuling I, Kootte RS, et al Impact of oral vancomycin on gut microbiota, bile acid metabolism, and insulin sensitivity. J Hepatol. 2014;60(4):824–31. Epub 2013/12/10. S0168-8278(13)00837-4 [pii] 10.1016/j.jhep.2013.11.034 . 24316517

[36] Bech-Nielsen GV, Hansen CH, Hufeldt MR, Nielsen DS, Aasted B, Vogensen FK, et al Manipulation of the gut microbiota in C57BL/6 mice changes glucose tolerance without affecting weight development and gut mucosal immunity. Res Vet Sci. 2012;92(3):501–8. Epub 2011/05/06. S0034-5288(11)00128-7 [pii] 10.1016/j.rvsc.2011.04.005 . 21543097

[37] Murphy EF, Cotter PD, Hogan A, O'Sullivan O, Joyce A, Fouhy F, et al Divergent metabolic outcomes arising from targeted manipulation of the gut microbiota in diet-induced obesity. Gut. 2013;62(2):220–6. Epub 2012/02/22. gutjnl-2011-300705 [pii] 10.1136/gutjnl-2011-300705 . 22345653

[38] Hernandez E, Bargiela R, Diez MS, Friedrichs A, Perez-Cobas AE, Gosalbes MJ, et al Functional consequences of microbial shifts in the human gastrointestinal tract linked to antibiotic treatment and obesity. Gut Microbes. 2013;4(4):306–15. Epub 2013/06/21. 25321 [pii] 10.4161/gmic.25321 23782552 PMC3744515

[39] Arumugam M, Raes J, Pelletier E, Le Paslier D, Yamada T, Mende DR, et al Enterotypes of the human gut microbiome. Nature. 2011;473(7346):174–80. Epub 2011/04/22. 10.1038/nature09944 21508958 PMC3728647

[40] Chevrot R, Carlotti A, Sopena V, Marchand P, Rosenfeld E. Megamonas rupellensis sp. nov., an anaerobe isolated from the caecum of a duck. Int J Syst Evol Microbiol. 2008;58(Pt 12):2921–4. Epub 2008/12/09. 58/12/2921 [pii] 10.1099/ijs.0.2008/001297-0 . 19060083

[41] Sakamoto M, Takagaki A, Matsumoto K, Kato Y, Goto K, Benno Y. Butyricimonas synergistica gen. nov., sp. nov. and Butyricimonas virosa sp. nov., butyric acid-producing bacteria in the family 'Porphyromonadaceae' isolated from rat faeces. Int J Syst Evol Microbiol. 2009;59(Pt 7):1748–53. Epub 2009/06/23. ijs.0.007674–0 [pii] 10.1099/ijs.0.007674-0 . 19542124

[42] Nagai F, Morotomi M, Watanabe Y, Sakon H, Tanaka R. Alistipes indistinctus sp. nov. and Odoribacter laneus sp. nov., common members of the human intestinal microbiota isolated from faeces. Int J Syst Evol Microbiol. 2010;60(Pt 6):1296–302. Epub 2009/08/12. ijs.0.014571–0 [pii] 10.1099/ijs.0.014571-0 . 19667375

[43] Cani PD BR, Knauf C, Waget A, Neyrinck AM, Delzenne NM, Burcelin R. Changes in gut microbiota control metabolic endotoxemia-induced inflammation in high-fat diet-induced obesity and diabetes in mice. Diabetes. 2008. 10.2337/db07-1403 18305141

[44] Vrieze A, Van Nood E, Holleman F, Salojarvi J, Kootte RS, Bartelsman JF, et al Transfer of intestinal microbiota from lean donors increases insulin sensitivity in individuals with metabolic syndrome. Gastroenterology. 2012;143(4):913–6.e7. Epub 2012/06/26. 10.1053/j.gastro.2012.06.031 . 22728514

[45] Delzenne NM, Cani PD, Everard A, Neyrinck AM, Bindels LB. Gut microorganisms as promising targets for the management of type 2 diabetes. Diabetologia. 2015;58(10):2206–17. Epub 2015/08/01. 10.1007/s00125-015-3712-7 [pii]. . 26224102

[46] De Vadder F, Kovatcheva-Datchary P, Goncalves D, Vinera J, Zitoun C, Duchampt A, et al Microbiota-generated metabolites promote metabolic benefits via gut-brain neural circuits. Cell. 2014;156(1–2):84–96. Epub 2014/01/15. S0092-8674(13)01550-X [pii] 10.1016/j.cell.2013.12.016 . 24412651

[47] Perry RJ, Peng L, Barry NA, Cline GW, Zhang D, Cardone RL, et al Acetate mediates a microbiome-brain-beta-cell axis to promote metabolic syndrome. Nature. 2016;534(7606):213–7. Epub 2016/06/10. 10.1038/nature18309 27279214 PMC4922538

[48] Tolhurst G, Heffron H, Lam YS, Parker HE, Habib AM, Diakogiannaki E, et al Short-chain fatty acids stimulate glucagon-like peptide-1 secretion via the G-protein-coupled receptor FFAR2. Diabetes. 2012;61(2):364–71. Epub 2011/12/23. db11-1019 [pii] 10.2337/db11-1019 22190648 PMC3266401

[49] Zhang X, Shen D, Fang Z, Jie Z, Qiu X, Zhang C, et al Human gut microbiota changes reveal the progression of glucose intolerance. PLoS One. 2013;8(8):e71108 Epub 2013/09/10. 10.1371/journal.pone.0071108 PONE-D-13-14154 [pii]. 24013136 PMC3754967

