# Supplementary figures and images for: H. pylori eradication with antibiotic treatment causes changes in glucose homeostasis related to modifications in the gut microbiota
Source: PLoS One. 2019 Mar 14;14(3):e0213548. doi: 10.1371/journal.pone.0213548 (PMC6417676; doi:10.1371/journal.pone.0213548)

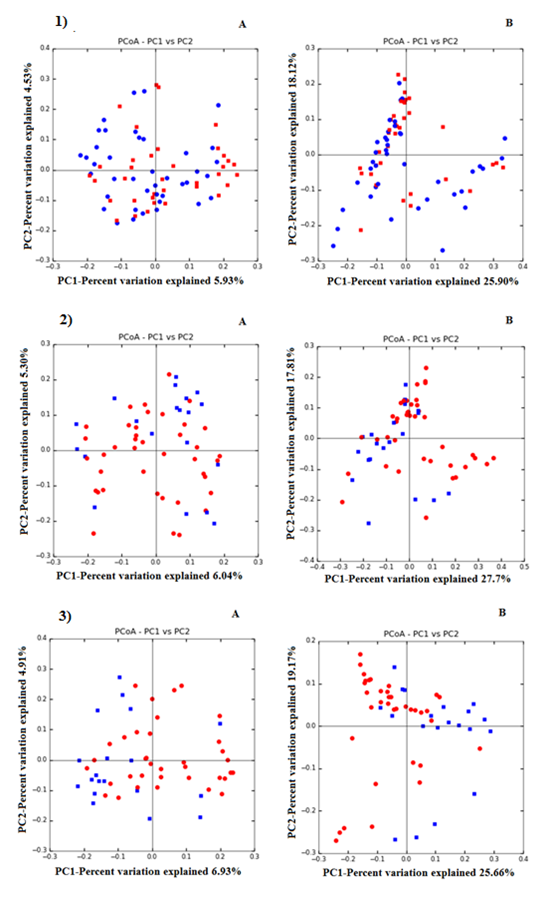

Supplement: S1 Fig — Clustering of fecal bacterial communities according to the different study groups by principal coordinate analysis (PCoA) using unweighted (A) and weighted (B) UniFracdistances. Each point corresponds to a community coded according to the patients and controls group: 1) Pre- (blue dot) vs. Post-H. pylori eradication (red squared),2) Controls(redsquared) vs. Post-H. pylori eradication (red dot). 3) (bluesquared) vs. Pre-H. pylori eradication (red dot).The percentage of variation explained by the plotted principal coordinates is indicated on the axes. (TIF) [file pone.0213548.s001.tif]

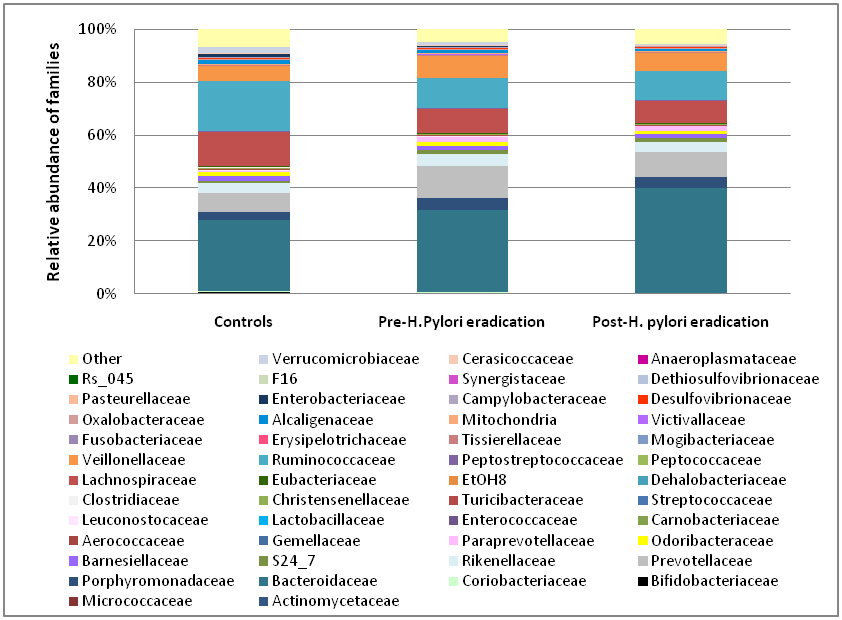

Supplement: S2 Fig — Other: sequences unassigned to OTU (TIF) [file pone.0213548.s002.tif]

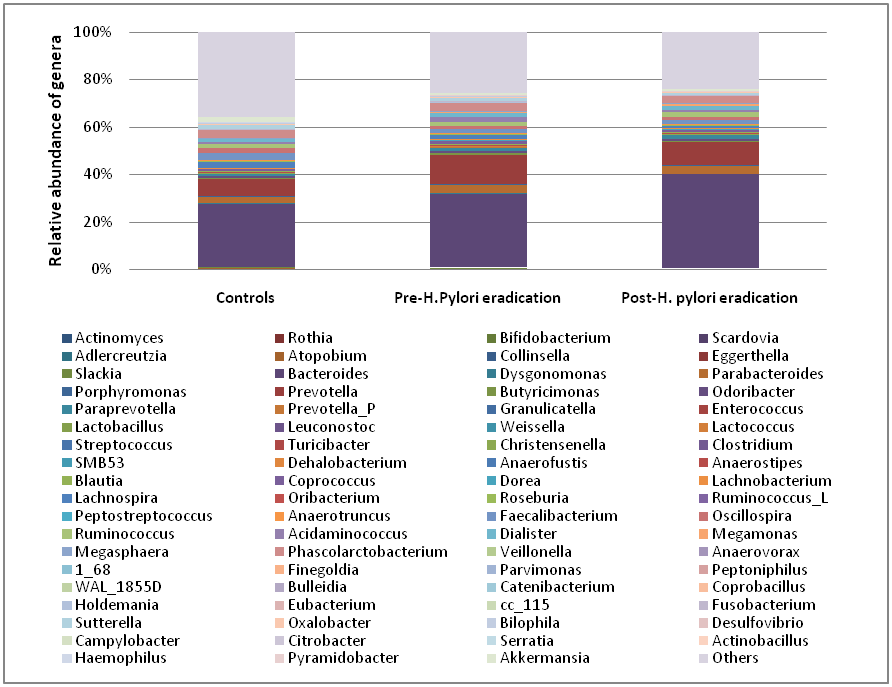

Supplement: S3 Fig — Other: sequences unassigned to OTU. (TIF) [file pone.0213548.s003.tif]

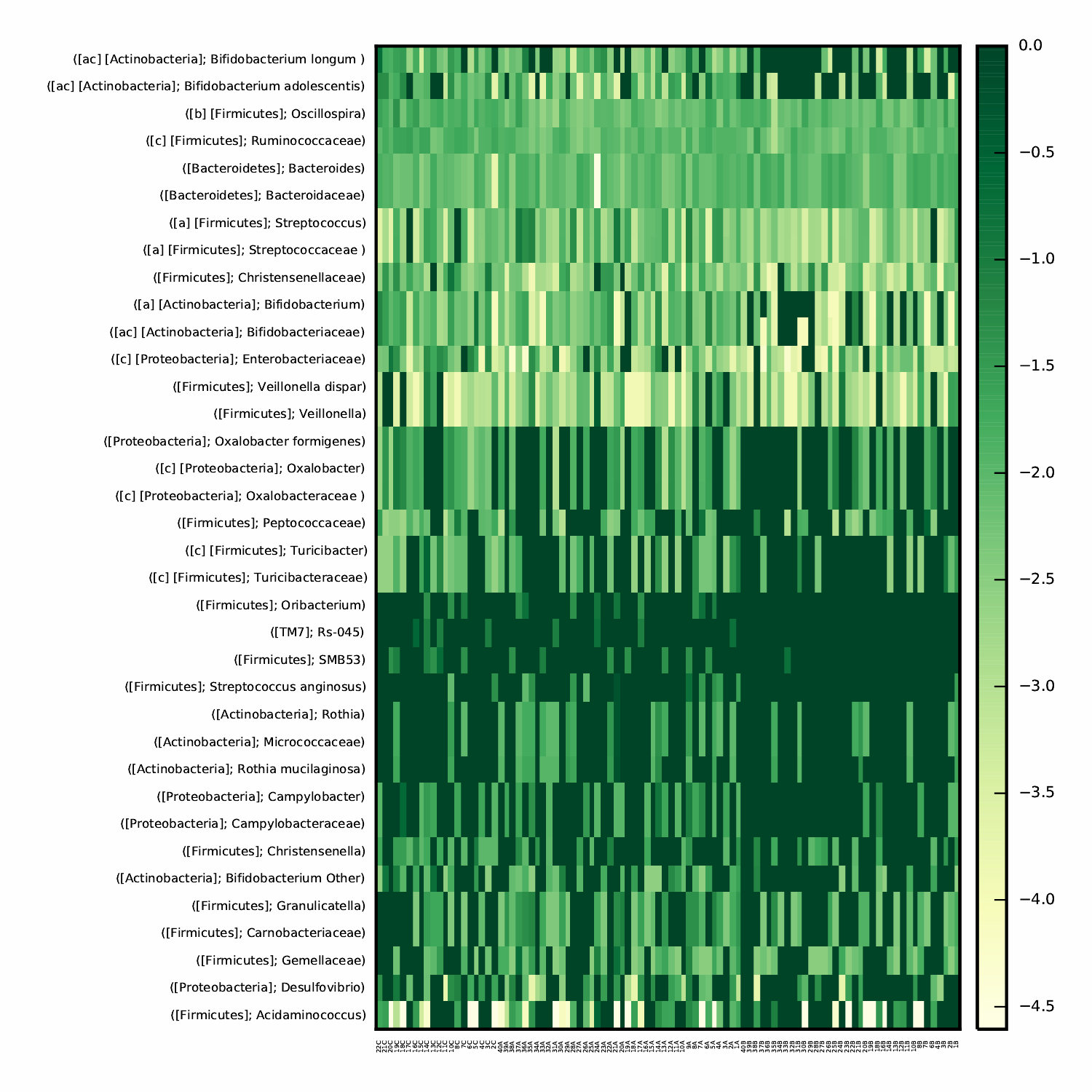

Supplement: S4 Fig — Significant bacterial taxa among groups are depicted. Wilcoxon’s signed-rank test was used in comparing pre and post-H. pylori eradication. U de Mann-Whitney was used to compare the unpaired-samples. Moreover, significant taxa after a multiple FRD correction (P<0.05) are indicated as: [a]: Pre- vs. Post-H. pylori eradication; [b]: control vs. Pre-H. Pylori eradication; [c]: Control vs. Post-H. pylori eradication. (TIFF) [file pone.0213548.s004.tiff]
